# Book Review: Negotiating Identity in Modern Foreign Language Teaching

**DOI:** 10.3389/fpsyg.2020.615120

**Published:** 2020-11-27

**Authors:** Shanshan Wang

**Affiliations:** Department of Language and Culture, Tianhua College, Shanghai Normal University, Shanghai, China

**Keywords:** teacher identity, foreign language teaching, language professionals, teacher cognition, teacher agency, language teacher education, perception of self

To truly understand language teaching and learning, we need to “understand teachers and to appreciate the professional, cultural, social, and individual identities which they claim or which are assigned to them” (p. 204). The understanding of modern language (MFL) teachers' lifeworld has been central to that end. *Negotiating Identity in Modern Foreign Language Teaching*, edited by Matilde Gallardo, is a timely volume offering profound insights into understanding MFL teachers' voices and their sense of agency in shaping their professional lives in the UK and beyond. Consisting of an introductory chapter and two body parts in another eight chapters, it is a revealing collection to illuminate many different possible ways in which we could give voice to, make sense of, and nurture MFL teachers' telling their stories of becoming and being who they are as language professionals among other identities.

The first body part (Chapters 2–7) is a collection of partitioner perspective empirical research exploring teachers' professional identity and their lived experiences of teaching. As MFL teachers could be members of academic departments or affiliations as well as pluricultural and plurilingual individuals, their experiences of being migrant and/or as pluricultural and plurilingual individuals are critical to pinpoint the influence of social–political and demographic factors as they negotiate a sense of self. Chapter 2 explores the experiences and perceptions of transnational MFL teachers living and working in the UK highlighting the “dynamic, hybrid, and non-static” (p. 36) nature of their identities while it also shows the sense of agency of those MFL teachers in “managing their conflicting attitudes and values” in the challenging work conditions due to educational changes, societal uncertainties, and difficult conditions of service. Chapter 3 examines the under-represented female Russian MFL teachers' identity formation contextualized by the discourse of the profession and immigration, motherhood, and childcare through storytelling.

To explore the lived experiences of language teachers and in particular their sense of self, the next chapter positions the study within the commonly employed sociocultural and post-structuralist theory and the debate around Native Speaker vs. Non-native Speaker. The study reveals the complex, multiple, and shifting nature of language teachers' identity, and equally importantly finds that the seemingly contradictory experiences of being a language teacher as being someone else and multiple selves are actually “harmonized and energizing” (p. 86). The intermediary role of their lived experience as language teachers also stands out as it mediates between language, culture, and language learners.

The study in Chapter 5 continues to delve into the complex, shifting, and fluid nature of MFL teachers' identity with a focus on the interplay between personal and professional in the trajectory of language professionals unfolding their “nomadic stories” of professionalization. Becoming a language professional, as discussed in this chapter, is a non-linear journey with personal and professional knowledge intertwined and interrelated with each other across boundaries and across time and space. To empower MFL professionals to be active agents in developing their own professional identity on a more practical level, Chapter 6 outlines a replicable model in which MFL teachers can reify their identity as professionals through engaging with action research. The chapter followed proceeds to unveil the influence of communities of practice and affiliations in shaping MFL teachers' identity over time and space. Discussions suggest that communities of practice help to nurture confidence and clarity of their identity in the early stages of their career while teacher agency exerts stronger influence as their careers develop.

The two final chapters form the second body part of the book featuring personal reflections of four teachers' experiences of becoming MFL teachers exemplifying many of the findings and claims made in the seven previous chapters. Studies in the two chapters unveil “the complex, fluid relationship between language and identity in the MFL context” (p. 197) and highlight the resilience of teacher agency in making difficult choices and challenging conventional practices in their trajectory of becoming who they are as MFL professionals. Notably, they stress the significant role of early childhood learning experiences and critical incidents in early teaching career on later professional choices in terms of empowering their agency.

In light of the paramount importance of identity in MFL education, teacher education, and development and the growing interest in the complex dynamics between various contextual, social factors, and identity negation, the thorough and nuanced explorations in the volume will serve as informative, insightful, and intriguing references for scholars, researchers, teachers, and students interested in the matter. Although limited to the UK context, the study makes timely contributions to the existing literature body and has significant implications for pertinent research in the future worldwide on theoretical, methodological, and empirical levels.

Theoretically and methodologically, with a strong theorizing focus on sociocultural and post-structural theory, studies in this volume also stand out by exploring the field from diverse social science perspectives and approaches such as social identity theory, feminist theory, nomadic theory, lived experiences perspective, etc., which enables readers to view MFL teachers' identity formation through various and equally valid lens. Studies in this volume also evidence that practitioner inquiry and autobiographical narrative could aptly be served as powerful research tools to help bridge the gap between the practitioner and the researcher. The long-existed dichotomy of teacher-researcher can be softened to make it possible that the MFL professionals can be the researcher as well as the researched at the same time. Empirically, voices emerge from the nine chapters, and the pertinent discussions around shed light on how MFL professionals exercise their agency and resilience in shaping their professional lives. These voices once again remind us of, as (Nunan and Choi, 2010) put, “the centrality of the human story to qualitative research in terms of what the story is and how the story is told” (2020, p. 1).

With all the merits mentioned, readers worldwide may benefit more from it if it had encompassed empirical studies and narratives of MFL teachers beyond the UK context, as voices from those teachers may present immensely varied landscapes embedding diverse climate across geographical, linguistic, and sociocultural boundaries and spaces. As the famous philosopher Husserl (2001) argues, all phenomena and objects invite us to delve deeper into and explore further. The volume may inspire other researchers to make further methodologically as well as theoretically informed exploration of this worthwhile but under-researched field of MFL teacher identity.

## Author Contributions

The author confirms being the sole contributor of this work and has approved it for publication.

## Conflict of Interest

The author declares that the research was conducted in the absence of any commercial or financial relationships that could be construed as a potential conflict of interest.
